# Impact of Coal Mining on Self-Rated Health among Appalachian Residents

**DOI:** 10.1155/2015/501837

**Published:** 2015-07-09

**Authors:** Shannon M. Woolley, Ada O. Youk, Todd M. Bear, Lauren C. Balmert, Evelyn O. Talbott, Jeanine M. Buchanich

**Affiliations:** ^1^Department of Biostatistics, Graduate School of Public Health, University of Pittsburgh, 130 De Soto Street, Pittsburgh, PA 15261, USA; ^2^Department of Behavioral and Community Health, Graduate School of Public Health, University of Pittsburgh, 130 De Soto Street, Pittsburgh, PA 15261, USA; ^3^Department of Epidemiology, Graduate School of Public Health, University of Pittsburgh, 130 De Soto Street, Pittsburgh, PA 15261, USA

## Abstract

*Objective*. To determine the impact of coal mining, measured as the number of coal mining-related facilities nearby one's residence or employment in an occupation directly related to coal mining, on self-rated health in Appalachia. *Methods*. Unadjusted and adjusted ordinal logistic regression models calculated odds ratio estimates and associated 95% confidence intervals for the probability of having an excellent self-rated health response versus another response. Covariates considered in the analyses included number of coal mining-related facilities nearby one's residence and employment in an occupation directly related to coal mining, as well as potential confounders age, sex, BMI, smoking status, income, and education. *Results*. The number of coal mining facilities near the respondent's residence was not a statistically significant predictor of self-rated health. Employment in a coal-related occupation was a statistically significant predictor of self-rated health univariably; however, after adjusting for potential confounders, it was no longer a significant predictor. *Conclusions*. Self-rated health does not seem to be associated with residential proximity to coal mining facilities or employment in the coal industry. Future research should consider additional measures for the impact of coal mining.

## 1. Introduction

The Appalachian Region is a 205,000-square-mile area that follows the spine of the Appalachian Mountains and includes all of West Virginia and parts of 12 other states [1]. Residents of Appalachia and other rural regions in the United States have higher rates of poverty, lower education levels, and more limited access to health care [2]. Many Appalachian communities also bear the burden of environmental exposure to toxicants from coal mining [3], chemical industries [4], metal refineries [5], and environmental tobacco smoke [6]. Specifically, residents in Appalachia are at an increased risk for diseases with environmental components—such as heart disease [7, 8], cancer [9], diabetes [10], and obesity [11]—compared with other ethnic groups or those living in nonrural areas [12]. While numerous studies have confirmed that health disparities exist in Appalachia, there have been conflicting findings across studies as to whether these health disparities stem from the socioeconomic disadvantages found in the region and/or from environmental impacts [3, 13–17].

Coal mining is one of the major economic industries for eight Appalachian states (Alabama, Kentucky, Maryland, Ohio, Pennsylvania, Tennessee, Virginia, and West Virginia) [18]. The area's need and support for coal mining make it important to understand any potential health risks posed to those involved and those living nearby. Residents of Appalachian coal mining communities have expressed concerns regarding illnesses after reported exposure to contaminated air and water from coal mining activities [19]; however, it is unclear whether living near coal mining sites negatively affects health [15, 20]. Quantitative research on the relationship between residential proximity to coal mining sites and health consequences has been limited to studies in Great Britain and to a narrow range of respiratory illnesses. The studies found that residential proximity to coal mining sites was associated with elevated levels of particulate matter [21] and increased symptoms of respiratory morbidity [22].

The use of self-rated health (SRH) as a predictor of mortality is well-established [23]. Twenty-seven international and US-based community studies show impressively consistent findings for SRH as an independent predictor of mortality [24]. The purpose of this analysis is to investigate the impact of coal mining, measured as the number of coal mining-related facilities nearby one's residence or employment in an occupation directly related to coal mining, on SRH in Appalachia.

## 2. Methods

### 2.1. Sample

The sample was drawn primarily from 10 counties of interest (five coal mining and five noncoal mining): coal: Boone, WV; Logan, WV; Mingo, WV; Monongalia, WV; and Raleigh, WV; noncoal: Berkeley, WV; Calhoun, WV; Tyler, WV; Yancey County, NC; and Cocke County, TN. A total sample of 9000 telephone numbers, drawn from a pool of all landline and cellular telephone phone numbers in the relevant area codes, was purchased from Genesys Marketing Systems Group (MSG) (http://www.m-s-g.com/web/index.aspx). Numbers were prescreened by Genesys MSG to remove nonworking or nonresidential numbers; 5054 numbers were available to be called, with a target sample size of 400–500, chosen as the target sample size because this was intended as a demonstration project.

### 2.2. Survey

The survey consisted of about 120 questions, collecting demographic, behavioral, and socioeconomic risk factors, attitudes about community and environmental satisfaction, access to health care, and physical and emotional health information. It was programmed in Ci3 (a questionnaire authoring application) and loaded into Win-Cati 5.0, a computer assisted telephone interviewing (CATI) program using the Windows platform. The Win-Cati system enabled supervisors to ensure that phone numbers were called on schedule and allowed for direct data entry, reducing the likelihood of data entry errors. Numbers were called at different times during the day, evening, and weekend call periods to increase the likelihood of contact, with an average of 2.2 call attempts to each number.

### 2.3. Statistical Analyses

SRH was measured using responses from the survey question, “Would you say that in general your health is…,” rated on a 5-point Likert scale from 1 (excellent) to 5 (poor) (question 53). We independently compared SRH responses for groups expected to have different levels of exposure to coal mining. The first comparison was based on the self-reported number of coal mining facilities nearby that person's home or the number of “yes” responses to questions which asked participants whether or not they lived near the following facilities (questions 44–49): underground continuous coal mine, underground longwall coal mine, mountaintop removal coal mine, surface coal mine, impoundment pond, or coal preparation facility. For example, if someone lived near all of the possible coal mining facilities, they were considered to live near six coal facilities. The second comparison was based on the survey question, which asked participants, “Are you or have you been employed in an occupation directly related to coal mining?” (question 96). Graphical representations were created to compare and contrast SRH status for these questions. Because we were interested in SRH as related to coal mining, a predominantly male occupation, we examined the descriptive characteristics by gender and ran gender-stratified and not stratified models.

We used ordinal logistic regression to investigate whether the number of coal facilities nearby and/or having an occupation directly related to coal mining are predictors of SRH after adjusting for age, sex, and other potential confounders. Other potential confounders were included in adjusted multivariable models for SRH if the potential confounder had univariate statistical significance of *p* < 0.15. Due to the small number of females with work in coal mines, we restricted those analyses to males only. Score tests were used to assess the proportional odds assumption for all models. For the male only models, SRH was collapsed into three categories (excellent, very good + good, and fair + poor) in order to satisfy the assumption of proportional odds. Statistical significance for all multivariable models was assessed as *p* < 0.05. This study was approved by the University of Pittsburgh Institutional Review Board. Respondents were not provided an incentive for their participation.

## 3. Results

**Figure 1:**
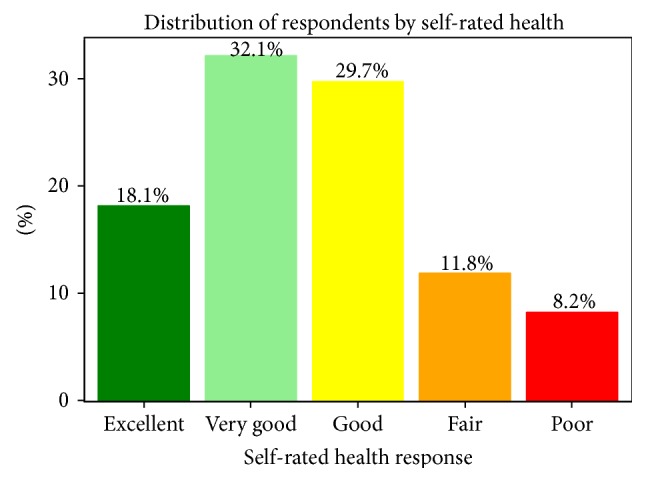


Each telephone number in the sample was called at least once. Numbers called more than once were called back at different times during the day, evening, and weekend call periods to increase the likelihood of contact. An average of 2.2 call attempts were made to each number. A total of 415 interviews were completed. Using American Association for Public Opinion Research (AAPOR) standard definitions [25], the cooperation rate for the study was 34% (415/1235) and the response rate was 10.9% (415/3793), with an average interview length of 28.6 minutes.  Figure 1 shows the distribution of responses for the outcome variable of interest, SRH. Approximately half of the respondents reported their health as excellent or very good.

Table 1 shows, by sex, the demographic characteristics of the sample. Males were slightly more likely than females to be every day smokers, were less likely to earn less than $25,000, were more likely to have less than a HS degree education, and were more likely to live near a coal mining facility or be employed in a coal-related occupation.

As shown in Table 2, age, BMI, smoking status, income, and education were highly statistically significantly related to SRH. As age and BMI increased, respondents were more likely to report poorer SRH. Lower incomes and less education were also negatively associated with SRH. Employment in a coal-related occupation was also statistically significantly negatively associated with SRH (*p* = 0.04). This relationship was more pronounced in males (*p* = 0.01).

The number of coal mining facilities near the respondent's residence was not a statistically significant predictor of SRH. Adjustment by the covariates age, sex, BMI, smoking status, income, and level of education did not change the association between SRH and number of mining facilities nearby (Table 3). However, the model estimates suggest a potentially linear relationship, indicating that as the number of coal mining facilities nearby increases, the probability of poorer SRH increases. The effect of living nearby 5 or 6 coal facilities versus living nearby 0 coal facilities approached statistical significance (*p* = 0.08).

In the stratified models, the number of nearby coal facilities was not statistically significant for males or females (data not shown). In the males only model, BMI and smoking were statistically significant predictors of SRH (*p* < 0.05); in the females only model, age, BMI, smoking, income, and education were all statistically significant predictors of SRH (*p* < 0.05).

As shown in Table 4, after adjusting for age, sex, BMI, smoking status, income, and level of education, employment in a coal-related occupation was no longer a statistically significant predictor of SRH in males (*p* = 0.98). Higher BMI, smoking every day, and having a high school or less education were statistically significant predictors of poorer SRH.

## 4. Discussion

Compared to WV overall, the respondents of this survey were less likely to report their health as fair or poor (20% survey versus 25.2% WV) [26]; in fact, 50% of participants reported being in excellent or very good health. The percent of current smokers in this sample (22.9%) is lower than the WV average of 27.3% but still higher than the average for the US (19.0%) [26]. However, the median self-reported BMI (BMI = 28.5) is considered overweight. These results are similar to those found by Griffith et al. (2011) that self-reported health status in Appalachia was incongruent with current health behaviors [27]. In addition, the covariate factors of the sample participants are representative of the entire study population [28]. More males than females reported living near any coal facility, although the overall percent (45.5%) provided adequate discrimination to use number of coal facilities as a covariate in the models. A much higher percent of males worked in a coal-related occupation; as such, these models were stratified to analyze males separately.

In the univariable ordinal logistic models, SRH is also associated with the demographic/socioeconomic factors of age, income, and level of education, as well as the personal risk factors of obesity and smoking every day. The number of coal facilities near the respondent's residence was not associated with SRH. Employment in coal-related occupations was found to be negatively associated with SRH.

**Figure 2:**
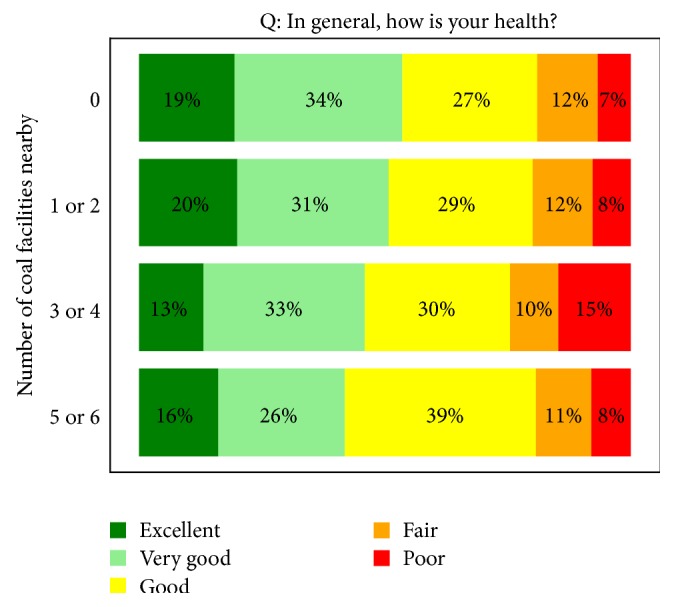


As shown in Figure 2, there does not appear to be a trend for SRH category and number of coal mining facilities nearby; however, there are notable differences between the mine groups. The percentage of respondents who reported “excellent” or “very good” health decreases slightly as the number of coal facilities nearby increases.

Zullig and Hendryx (2011) found that residents of mountaintop mining communities generally had poor self-reported health [29]. In our survey, a separate analysis of questions regarding proximity to mountaintop removal and surface coal mines (questions 46 and 47, resp.) found no association between SRH and living near a surface coal mine (univariable ordinal logistic regression model *p* = 0.92, data not shown). Age, BMI, smoking, income, and education were all highly statistically significantly associated with poorer SRH in the multivariable model. These factors seemed to affect SRH in females more than males.

We found no evidence of an association between SRH and employment in an occupation related to coal mining. However, the percentage of participants reporting a “poor” health status was 9% higher for those who are or have been employed in an occupation related to coal mining versus those who are/have not. In addition, approximately 62% of men with mining employment have an education level corresponding to “high school or less,” whereas the education levels of men without such employment are more evenly distributed. Thus, it is possible that, by including both of these variables in the model, the effect of coal-related employment on SRH is being masked by education level.

This study has some limitations. First, survey data can be prone to self-reporting bias, although SRH is a validated outcome [23]. The number of nearby coal mining facilities may not accurately reflect the effects of coal mining on the individual and, in some instances, respondents were unaware of the proximity of facilities. Future work should consider geospatial analysis using exact addresses and distances between coal mining facilities and residents' homes; however, these results are strengthened by the ability to control for individual-level covariates and exposures. After covariate adjustment, the results suggested a linear relationship between SRH and the number of coal mining facilities, but it was not found to be statistically significant. This could be due to the size of our sample. While we were able to detect statistically significant relationships between SRH and other covariates of interest, the number of interviews was relatively small. A larger sample size may be able to elucidate statistically significant relationships that this study was not, such as one between SRH and the number of coal facilities nearby. Additional studies should focus on the magnitude of this potential relationship using exact address information.

This study found no associations between SRH and residential proximity to coal mining facilities or employment in the coal industry. It did, however, provide support for the body of literature associating lower SRH with individual-level factors age, smoking status, income, and education.

## Figures and Tables

**Table 1:** Descriptive statistics [mean ± SD or *n* (%)] for covariates.

Covariate	Males (*n* = 195)	Females (*n* = 220)	Total (*n* = 415)
Age	54.0 ± 16.7	55.0 ± 17.5	54.5 ± 17.1
BMI	29.1 ± 5.7	27.8 ± 6.6	28.5 ± 6.2
Smoking status			
Not at all	151 (77.4)	169 (76.8)	320 (77.1)
Some days	9 (4.6)	17 (7.7)	26 (6.3)
Every day	35 (18.0)	34 (15.5)	69 (16.6)
Income level			
Under $25,000	42 (21.5)	70 (31.8)	112 (27.0)
$25,000 up to $49,999	42 (21.5)	46 (20.9)	88 (21.2)
$50,000 up to $74,999	43 (22.1)	39 (17.7)	82 (19.8)
$75,000 or more	68 (34.9)	65 (29.6)	133 (32.1)
Education level			
High school or less	90 (46.2)	80 (36.4)	170 (41.0)
Some college/associates	44 (22.6)	72 (32.7)	116 (28.0)
Bachelors or more	61 (31.3)	68 (30.9)	129 (31.1)
Number of coal facilities nearby			
0	82 (42.1)	144 (65.5)	226 (54.5)
1 or 2	33 (16.9)	32 (14.6)	65 (15.7)
3 or 4	36 (18.5)	26 (11.8)	62 (14.9)
5 or 6	44 (22.6)	18 (8.2)	62 (14.9)
Employed in coal-related occupation			
No	111 (56.9)	204 (92.7)	315 (75.9)
Yes	84 (43.1)	16 (7.3)	100 (24.1)

**Table 2:** Ordinal logistic regression univariable models for association between SRH and covariates.

Covariate	OR	95% Wald confidence limits		*p* value
Age	0.98	0.97	0.99	<0.01^*∗∗*^
BMI	0.92	0.89	0.95	<0.01^*∗∗*^
Sex				0.77
Female	1.00			
Male	1.05	0.75	1.49	
Smoking status				<0.01^*∗∗*^
Not at all	1.00			
Some days	0.81	0.39	1.65	
Every day	0.36	0.23	0.59	
Income level				<0.01^*∗∗*^
Under $25,000	1.00			
$25,000 up to $49,999	1.65	1.00	2.73	
$50,000 up to $74,999	4.16	2.45	7.08	
$75,000 or more	3.77	2.36	6.04	
Education level				<0.01^*∗∗*^
High school or less	1.00			
Some college/associates	1.35	0.88	2.07	
Bachelors or more	4.79	3.08	7.43	
Number of coal mines nearby				0.40
0	1.00			
1 or 2	0.94	0.57	1.54	
3 or 4	0.68	0.41	1.13	
5 or 6	0.75	0.45	1.23	
Employed in coal-related occupation				0.04^*∗*^
No	1.00			
Yes	0.65	0.44	0.98	

^*∗*^Statistically significant at *p* < 0.05.

^*∗∗*^Statistically significant at *p* < 0.01.

**Table 3:** Ordinal logistic regression model for association between SRH, number of nearby coal facilities, and covariates.

Covariate	OR	95% Wald confidence limits		*p* value
Age	0.98	0.97	0.99	<0.01^*∗∗*^
BMI	0.91	0.88	0.94	<0.01^*∗∗*^
Sex				0.20
Female	1.00			
Male	1.29	0.87	1.92	
Smoking status				<0.01^*∗∗*^
Not at all	1.00			
Some days	0.96	0.45	2.03	
Every day	0.37	0.21	0.64	
Income level				<0.01^*∗∗*^
Under $25,000	1.00			
$25,000 up to $49,999	1.23	0.70	2.14	
$50,000 up to $74,999	2.73	1.52	4.90	
$75,000 or more	2.19	1.26	3.81	
Education level				<0.01^*∗∗*^
High school or less	1.00			
Some college/associates	1.11	0.69	1.78	
Bachelors or more	2.52	1.51	4.21	
Number of coal facilities nearby				0.31
0	1.00			
1 or 2	0.91	0.53	1.55	
3 or 4	0.74	0.43	1.28	
5 or 6	0.60	0.34	1.06	

^*∗∗*^Statistically significant at *p* < 0.01.

**Table 4:** Ordinal logistic regression model for association between SRH, work in coal mining, and covariates (males only).

Covariate	OR	95% Wald confidence limits		*p* value
Age	0.99	0.97	1.01	0.23
BMI	0.90	0.85	0.95	<0.01^*∗∗*^
Smoking status				0.01^*∗*^
Not at all	1.00			
Some days	0.80	0.19	3.39	
Every day	0.27	0.11	0.65	
Income level				0.27
Under $25,000	1.00			
$25,000 up to $49,999	0.79	0.31	1.99	
$50,000 up to $74,999	1.84	0.73	4.61	
$75,000 or more	1.46	0.61	3.50	
Education level				0.03^*∗*^
High school or less	1.00			
Some college/associates	2.23	1.00	4.95	
Bachelors or more	2.73	1.18	6.30	
Employed in coal-related occupation				0.98
No	1.00			
Yes	0.99	0.52	1.88	

^*∗*^Statistically significant at *p* < 0.05.

^*∗∗*^Statistically significant at *p* < 0.01.
